# Potential Biomarkers of Peripheral and Central Fatigue in High-Intensity Trained Athletes at High-Temperature: A Pilot Study with *Momordica charantia* (Bitter Melon)

**DOI:** 10.1155/2020/4768390

**Published:** 2020-06-08

**Authors:** Jae-Jun Kwak, Jang Soo Yook, Min-Seong Ha

**Affiliations:** ^1^Department of National Defense Technology, Woosuk University, Daehak-ro 66, Jincheon-eup Jincheon-gun, Chungcheongbuk-do 27841, Republic of Korea; ^2^Center for Functional Connectomics, Korea Institute of Science and Technology (KIST), Hwarang-ro 14-gil 5, Seongbuk-gu, Seoul 02792, Republic of Korea; ^3^Division of Sports Neuroscience, Advanced Research Initiative for Human High Performance (ARIHHP), Faculty of Health and Sport Sciences, University of Tsukuba, 1-1-1 Tennoudai, Tsukuba, Japan; ^4^Laboratory of Exercise Biochemistry and Neuroendocrinology, Faculty of Health and Sports Sciences, University of Tsukuba, 1-1-1 Tennoudai, Tsukuba, Japan

## Abstract

Among potent dietary supplements, *Momordica charantia*, commonly called bitter melon, has various biological effects, such as antioxidant and anti-inflammatory effects, and improves energy metabolism and fatigue recovery. However, it is unknown whether *Momordica charantia* extract (MCE) induces antifatigue effects during exercise training in high-temperature environments. This study aimed at investigating the efficacy of MCE by examining 10 male tennis players consuming 100 mL MCE/dose (6 times a day over 4 weeks) during the summer training season. Peripheral (ammonia and uric acid) and central (serotonin, dopamine, and prolactin) fatigue parameters were measured before and after MCE consumption; before, during, and after exercise; and the next morning. After consuming MCE supplements, ammonia levels were higher during and after exercise and recovered the next morning, whereas uric acid levels did not change at any time point. Serotonin levels were lower during exercise. Dopamine levels were higher, especially during exercise. Prolactin levels were lower at all time points, especially during and after exercise. Although high-intensity training in a hot environment causes accumulation of fatigue-related metabolites, our results indicate that 4 weeks of MCE intake positively influenced fatigue parameters, suggesting that MCE can efficiently combat fatigue.

## 1. Introduction

Recent studies have reported diverse approaches to modulate athletes' physical condition and improve performance, such as the use of supplements derived from a variety of sources [1, 2]. In general, the main purposes of supplements are to improve immune function and prevent disease as well as to relieve dizziness, muscle damage, and cramps [3]. For athletes, in particular, supplements may relieve thirst during high-intensity training, supply moderate energy, and improve exercise performance, training adaptations, and fatigue resistance [4, 5]. In fact, the American College of Sports Medicine recommends taking supplements when exercising for longer than 1 hour, as they can help improve physiological function and maintain athletic performance during competitions [6].

Of the newer supplements being explored, there has been a focus on the use of natural plant extracts as added ingredients to sports drinks [7–9]. One of these extracts is from *Momordica charantia* (*M. charantia*), an annual vine of the gourd family (Cucurbitaceae) also called bitter melon owing to the bitter taste of the unripe fruit. It is used in highly nutritious drinks and as a medicinal plant in China and Japan because of its antioxidant activity, antidiabetic activity, and anti-inflammatory effect for improving human health [10–13]. *Momordica charantia* contains phytosterol glycosides, amino acids, galacturonate, citrulline, saponin, vitamin B1, beta carotene, soluble fibers, and potassium and is especially abundant in minerals, such as K, Ca, Mg, and Fe, as well as in vitamin C, which plays an important role in energy conversion [14, 15]. In addition, previous in vivo studies showed that *M. charantia* extract (MCE) from bitter melon exhibits physiological effects on energy metabolism via activation of activated AMP-activated protein kinase [16, 17] and also enhanced exercise performance in exhausted conditions with improved fatigue-related parameters such as lactate and ammonia [18]. These evidences lead us to postulate that MCE may affect fatigue resistance in trained athletes.

Fatigue due to continuous exercise is broadly divided into peripheral fatigue and central fatigue [19]. Peripheral fatigue decreases physical function owing to stimuli caused by a long period of exercise and refers specifically to fatigue in active muscles [20]. During peripheral fatigue, muscles have difficulty continuing to contract owing to depletion of creatine phosphate, neuromuscular signal transmitters, and glycogen [20, 21]. Central fatigue is fatigue that is perceived in the central nervous system (CNS) and is caused by changes in central neurotransmitters, such as serotonin, dopamine, and prolactin [22, 23]. Central fatigue symptoms manifest as psychological loss of motivation, loss of appetite, sleepiness, and reduced psychological alertness [24]. In high-temperature environments, the response of the CNS is closely related to central fatigue symptoms despite sufficient energy sources [25].

When training in a hot and humid environment, athletes sweat excessively, and the loss of moisture can cause physiological injury due to dehydration and exhaustion [26]. If water intake is insufficient, the blood becomes concentrated, resulting in hyperosmolarity, reduced plasma volume, and reduced blood flow, which in turn impair the release of heat from the body, causing heat retention [27]. Ultimately, loss of moisture through sweating leads to dehydration, reduced energy metabolism and exercise performance, and increased heat production in the body [28]. In addition, glycogen is more readily utilized as an energy source, resulting in an increased production of the byproducts that cause fatigue [29]. Thus, athletes performing high-intensity training in hot environments show high levels of peripheral and central fatigue, and it is important to find methods to alleviate the subsequent symptoms to ensure that athletes stay healthy and perform optimally.

The aim of our study was to determine the effects of regular intake of MCE on fatigue parameters in tennis athletes participating in a summer training program. To our knowledge, this was the first pilot study to analyze fatigue-related blood biomarkers in athletes who consumed MCE as a supplement. We hypothesized that tennis players consuming MCE on a regular basis would experience delayed fatigue and improved post-fatigue recovery measured by central and peripheral fatigue parameters during 4 weeks of high-intensity training in a high-temperature environment.

## 2. Materials and Methods

### 2.1. Participants

The subjects in our study were male tennis players enrolled at Pusan National University, South Korea (Table 1). Prior to the study, the subjects were thoroughly informed of the research aims and intentions, and written consent forms were collected. The study plan adheres to the guidelines and ethical principles of the Helsinki Declaration and was approved by the Pusan National University institutional review board (PNU IRB/2017_72_HR). The subjects underwent physical examination and testing as well as a medical interview by a physician to verify that they had no injuries or neurological or musculoskeletal disorders that could affect the experiment. Consumption of nutritional supplements and caffeinated beverages, alcohol, and antioxidant drugs was forbidden starting one month before the study, and intake of nonsteroidal anti-inflammatory drugs and supplements other than the MCE was restricted during the study period. The subjects participated in training on a regular basis, and additional personal exercise outside of team training was not permitted.

### 2.2. Study Design

This pilot study involving 10 tennis players investigated the effects of MCE on peripheral and central fatigue parameters. The subjects consumed MCE during 4 weeks of summer training. MCE was consumed 6 times/day at 100 mL/dose. Three doses were administered, with one dose each before, during, and after the exercise both in the morning and afternoon, with a total of 6 doses and 600 mL per day. Central fatigue parameters (serotonin, dopamine, and prolactin) and peripheral fatigue parameters (ammonia and uric acid) were measured before, during, and after exercise and the morning following exercise before consumption of MCE (week 0) and after consumption of MCE on a regular basis (week 4). Summer training was performed at 70–90% heart rate reserve, rating of perceived exertion 15–19 for aerobic exercise/tennis training and 60–85% one-repetition maximum, OMNI RES 6–8 for resistance exercise, for 120 minutes twice per day (morning: aerobic and resistance; afternoon: tennis), 5 days per week, for a total of 4 weeks, intending to improve physical condition and technique. For successful completion of the study, the researchers monitored, in real-time, not only the training program but also the subjects' lifestyle (camp accommodation, nutritional intake (analyzed by a computer-aided nutrition analysis program through a 24-hour recall method record; CAN Pro. Ver 3.0, The Korean Nutrition Society, Seoul, Korea; see Table 2), additional exercise, medical treatments, etc.).

### 2.3. MCE Sampling

*Momordica charantia* produced in South Korea, which contains biologically active compounds, such as polypeptides, saponins, alkaloids, flavonoids, phenolic acids, and carotenoids [30, 31], was used in this study. The basic constituents of MCE were analyzed by the Korea Food Research Institute and Pukyong National University Food Analysis Center, and the results are shown in Table 3. MCE samples were prepared as per methods verified in the previous studies of natural plants [7, 8, 32]. After adding 4 kg of fresh *M. charantia* and 6000 mL of hot water into an extractor, extraction was performed for 3 h at a temperature of 100°C and a pressure of 0.7 kg/cm^2^. MCE was sealed in small plastic bags in 100 mL portions and stored in a refrigerator.

### 2.4. Summer Training

The summer training program was devised by modifying a program designed by Kwak et al. [32]. In the summer training season, the average temperature reached 32.14°C ± 2.20°C. The athletes trained 120 min, twice/day (morning and afternoon), 5 days/week, for a total of 4 weeks, with the aim of improving physical condition and performance. Each training session consisted of 10 minutes each of warm-up and cool-down exercises and a 100-minute session of the main exercise. Throughout the training duration, athletes performed basic physical conditioning, specialized technique training, aerobic training, and weight training. The specific training contents are shown in Table 4. Exercise intensity was analyzed by measuring changes in heart rate using a heart rate monitor watch (Polar RS400sd; model APAC, 90026360; Polar, NY, USA) and Borg's rating of perceived exertion [33], one-repetition maximum, and OMNI resistance scale of perceived exertion (RES) [34]. All training sessions were supervised by the researchers.

### 2.5. Biochemical Analysis

Blood samples were collected before (week 0 of training) and after (week 4 of training) consuming MCE, making a total of 8 samples. After collecting 10 mL of blood from a forearm vein, the blood was centrifuged for 10 min at 3000 rpm using a centrifuge (Combi-514R; Hanil, Gimpo, Korea), and the plasma was isolated for analysis. The collected plasma was used to analyze peripheral fatigue (ammonia and uric acid) and central fatigue (serotonin, dopamine, and prolactin) parameters.

### 2.6. Sample Size Calculation

The optimal sample size was determined using the G-power version 3.1 Windows program (Kiel University, Kiel, Germany), based on a 0.25-point effect size (default), alpha level of *p* < 0.05, and 30% power [35]. The results indicated that 8 participants per group were needed. Assuming a dropout rate of 20%, the sample size was set to 10 participants.

### 2.7. Statistical Analysis

SPSS version 21.0 (IBM Corp., Chicago, IL, USA) was used for data processing. The mean and standard deviation were calculated for measured variables; repeated measure analyses of variance were performed to test the effects before and after MCE supplementation and between time points; and paired *t*-tests were performed to compare specific differences in fatigue indicators between time points. For all tests, a statistical significance level of *p* < 0.05 was used.

## 3. Results

### 3.1. Effect of MCE Supplementation on Peripheral Fatigue

**Figure 1:**
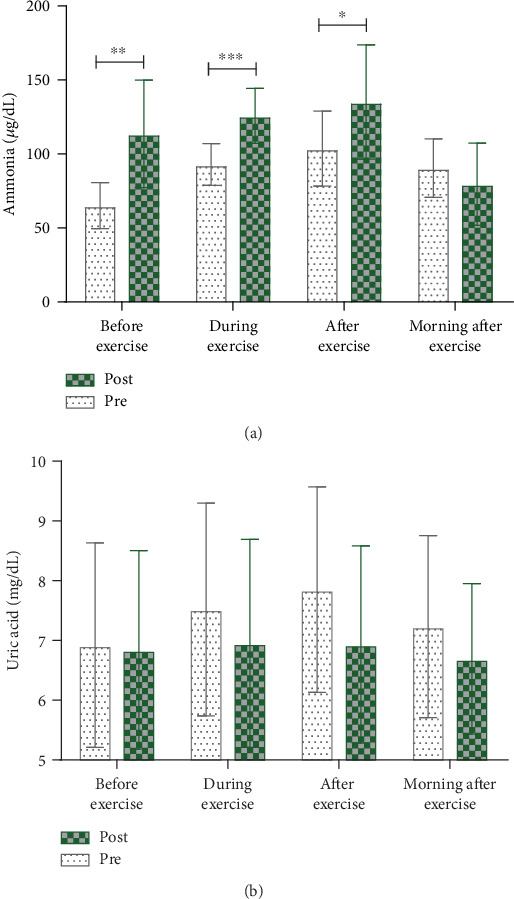
Comparison of peripheral fatigue parameters before and after MCE supplementation during the training program. (a) Both before and after MCE supplementation, ammonia levels increased from before, during, and after exercise and recovered by the next morning. Ammonia levels were higher after MCE supplementation and had a higher level of recovery. (b) Uric acid had a much lower increase after MCE supplementation than before MCE supplementation. ^∗^*p* < 0.05, ^∗∗^*p* < 0.01, ^∗∗∗^*p* < 0.001, compared to before-MCE supplementation values.

After MCE supplementation, ammonia levels in samples collected at week 4 were significantly higher before (*t* = −3.879, *p* ≤ 0.001), during (*t* = −4.464, *p* < 0.001), and immediately after exercise (*t* = −2.156, *p* < 0.05) than those in samples collected at week 0 (Table 5; Figure 1(a)). The MCE supplementation showed a significant interaction effect with ammonia response (time × supplementation effect: *F* = 5.250, *p* < 0.01; time effect: *F* = 6.317, *p* < 0.01; supplementation effect: *F* = 24.883, *p* ≤ 0.001) (Table 5). Analyses of changes in ammonia levels over time using samples collected before MCE supplementation revealed that ammonia levels were significantly higher during exercise (*t* = −5.272, *p* ≤ 0.001), immediately after exercise (*t* = −6.014, *p* < 0.001), and the morning after exercise (*t* = −3.063, *p* < 0.05) than before exercise. After MCE supplementation, ammonia levels showed an increasing trend during and immediately after exercise, but there were no significant differences when compared to the levels before exercise. Notably, after MCE supplementation, ammonia levels were significantly higher the morning after exercise (*t* = 2.344, *p* < 0.05) than before exercise (Table 5; Figure 1(a)).

Uric acid levels showed no significant differences before and after MCE supplementation at any of the four time points; however, the mean uric acid levels were lower after MCE supplementation (Table 5; Figure 1(b)). Regarding interaction effects, the time × supplementation interaction and supplementation effects were not significant, but there was a significant time effect (*F* = 6.003, *p* < 0.05). We analyzed the change in uric acid levels over time independently before and after MCE supplementation (Table 5; Figure 1(b)). Uric acid levels before MCE supplementation were significantly higher during (*t* = −3.947, *p* < 0.01) and immediately after exercise (*t* = −4.422, *p* < 0.01) than before exercise, and they also showed a trend toward decreased levels in samples collected the next morning, which was not statistically significant. After MCE supplementation, uric acid levels showed an increasing trend during exercise and immediately after exercise compared to those before exercise; however, the differences were not statistically significant. There was also no significant difference in the levels measured the next morning, but there was a trend toward lower uric acid levels before exercise.

### 3.2. Effect of MCE Supplementation on Central Fatigue

**Figure 2:**
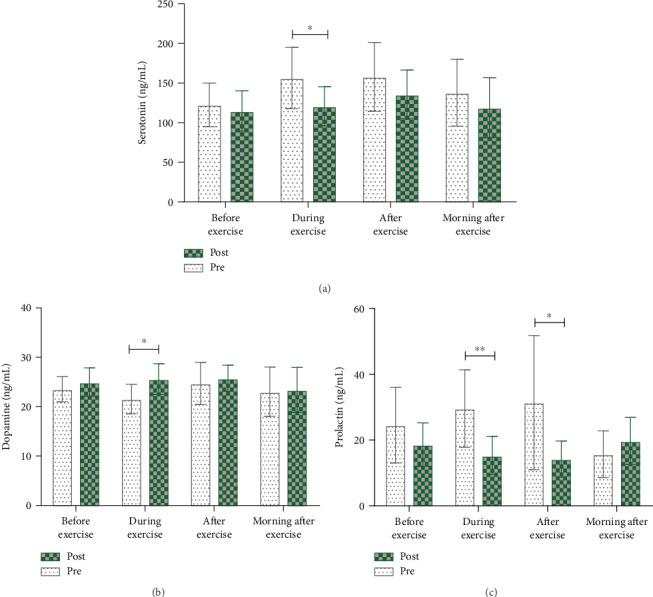
Comparison of central fatigue parameters before and after MCE supplementation during the training program. (a) Serotonin levels were lower after MCE supplementation than before MCE supplementation, particularly during exercise. (b) Dopamine levels were higher after MCE supplementation than before MCE supplementation and showed increased levels during and immediately after exercise but decreased to below rest levels in the recovery phase the next morning. (c) Prolactin levels were lower after MCE supplementation than before MCE supplementation and were higher in the recovery phase than in the rest phase. ^∗^*p* < 0.05, ^∗∗^*p* < 0.01 compared to before-MCE supplementation values.

There was an effect on the pattern of serotonin response after MCE supplementation (time × supplementation interaction effect: *F* = 3.134, *p* < 0.05; time effect: *F* = 12.791, *p* < 0.01; supplementation effect: *F* = 11.117, *p* < 0.01) (Table 6). Serotonin levels were lower after than before supplement use, and this difference was significant during exercise (*t* = 2.468, *p* < 0.05) (Table 6; Figure 2(a)). We also analyzed the changes in serotonin over time independently before and after MCE supplementation. Before MCE supplementation, serotonin levels were significantly higher during (*t* = −5.382, *p* < 0.001) and immediately after exercise (*t* = −3.978, *p* < 0.01) than those before exercise, and there was a trend toward decreased levels the next morning. After MCE supplementation, serotonin levels were lower than those before supplementation, and they were significantly higher during (*t* = −2.688, *p* < 0.05) and immediately after exercise (*t* = −6.432, *p* < 0.001) than those before exercise but were lower than those before exercise the next morning (Table 6; Figure 2(a)).

When we examined changes in dopamine, the time × supplementation interaction and time effects were not significant, but there was a significant supplementation effect (*F* = 5.186, *p* < 0.05) (Table 6). When we analyzed the differences in dopamine levels before and after MCE use, dopamine levels were higher after MCE supplementation than before MCE supplementation, and this difference was especially notable during exercise (*t* = −2.985, *p* < 0.01) (Table 6; Figure 2(b)). We also analyzed the changes in dopamine levels after time, independent of MCE supplementation. Before MCE supplementation, dopamine levels significantly decreased during exercise (*t* = 2.628, *p* < 0.05) compared to those before exercise, but they showed a trend toward slightly increased levels immediately after exercise. Dopamine levels measured the next morning were similar to those before exercise. Conversely, after MCE supplementation, there were no significant differences in dopamine levels between time points. However, there was a trend toward higher dopamine levels during and immediately after exercise, and the levels returned to normal before exercise the next morning (Table 6; Figure 2(b)).

When we examined the changes in prolactin levels with MCE supplementation, the time × supplementation interaction (*F* = 4.652, *p* < 0.05) and supplementation effects (*F* = 6.830, *p* < 0.05) were significant, but there was no significant time effect (Table 6). Analysis of differences in prolactin levels before and after MCE supplementation showed that prolactin levels after supplementation were lower and, in particular, were significantly lower during (*t* = 3.438, *p* < 0.01) and immediately after exercise (*t* = 2.577, *p* < 0.05) (Table 6; Figure 2(c)). We also examined the changes in prolactin levels over time, independent of MCE supplementation. Before MCE supplementation, prolactin showed a trend for increasing levels before, during, and after exercise, and then the levels lowered the next morning. However, after MCE supplementation, prolactin levels were significantly lower during (*t* = 4.613, *p* ≤ 0.001) and immediately after exercise (*t* = 2.842, *p* < 0.05) than those before exercise, and they returned to normal levels before exercise the next morning (Table 6; Figure 2(c)).

## 4. Discussion

For the first time, this study determined the efficacy of pre- and post-MCE supplementation on ammonia, uric acid, serotonin, dopamine, and prolactin as a biomarker for peripheral and central fatigue in high-intensity trained tennis players participating in a four-week training program at high-temperature environments. Exercising in high temperatures raises body temperature, causing fatigue and imbalance of heat production and loss. If the body temperature increases to 40°C, it causes not only fatigue but also reduced or impaired CNS function and can be life-threatening [26, 27]. Our findings showed that MCE supplementation positively changed the levels of fatigue parameters in blood, which indicated that MCE exhibited a potential antifatigue effect for alleviating exercise and high-temperature fatigue.

Ammonia, uric acid, and lactate dehydrogenase have been suggested as indicators to evaluate peripheral fatigue [36, 37]. A rapid removal of fatigue-related substances from the bloodstream is very important since it delays the decline in exercise performance and facilitates sustained exercise [19]. In this study, overall ammonia levels were higher after MCE supplementation than before MCE supplementation. Previous studies reported that, during high-intensity exercise, intramuscular acidification results in the release of ammonia [38]. Hence, the higher levels reported in this study can be interpreted as the result of increased exercise intensity, duration, and exposure to a high-temperature environment of summer training. It was expected that this would result in the degradation of protein and depletion of other sources to provide energy, thus producing ammonia as a by-product. Meanwhile, after MCE intake, ammonia significantly decreased the morning after exercise post the recovery period. It is believed that while moisture is lost owing to continual exercise in high temperatures, the electrolytes in MCE suppress hemoconcentration and loss of plasma volume while facilitating storage and supply of energy sources. In addition, MCE is believed to help protect against and prevent oxidative injury caused by the accumulation of ammonia during exercise. The results of this study related to symptoms of peripheral fatigue in human subjects are consistent with those of previous animal studies. For example, in one study of animals with induced hyperammonemia, MCE administration increased superoxide dismutase and catalase activity, inhibiting and improving oxidation [39]. In another animal study, MCE consumption reduced ammonia, lactate, serum creatine kinase, and blood urea nitrogen levels after exhaustion exercise [18]. Although our findings and previous reports suggest that ammonia levels as a fatigue-related parameter were positively modulated by MCE supplementation, the mechanisms underlying the effects of MCE remain. Recently, Chen et al. reported that hyperammonemia impairs energy metabolism by causing dysfunction of skeletal muscle mitochondria, which are repaired by catechin flavonoids through fatty acid oxidation and mitochondria function-associated gene expression [40]. Thus, the possible molecular mechanisms of MCE-mediated ammonia metabolism may be explained by the regulation of mitochondria respiration.

During muscle contraction, a large amount of adenosine triphosphate (ATP) is consumed, producing adenosine diphosphate [41]. Uric acid generally regenerates adenosine monophosphate into ATP through oxidative phosphorylation of purine nucleotides [41]. However, when ATP usage is high and there is a lack of oxygen for deoxidation to produce ATP, xanthine accumulates and is converted into uric acid by xanthine oxidase [42]. Since there is no urate oxidase in the human body, in order to maintain a state of equilibrium, around 700 mg of uric acid per day is excreted through the intestines and kidneys, and when this is insufficient, fatigue builds. In addition, uric acid exists in the form of sodium urate, and when the saturation limit (7.0 mg/dL) is exceeded, uric acid crystals form, causing inflammation [43]. Exercise increases uric acid levels via catabolism of purine nucleotides. According to previous research on changes in uric acid levels during exercise, the intensity is more important than exercise duration in increasing blood uric acid levels, and recovery from high uric acid levels can be optimized by consuming antioxidants, which remove free radicals [4, 43]. However, in the result of our study, there were no significant effects of MCE supplementation. Instead, our results suggest that ammonia, not uric acid, contributes to MCE-mediated antifatigue effects in the peripheral system.

Central fatigue is perceived in the CNS, resulting from changes in the levels of neurotransmitters, such as serotonin, dopamine, and prolactin [19, 44]. Central fatigue is associated with the difficulty in continuing exercise due to a psychological loss of motivation, loss of appetite, sleepiness, or impairment of psychological alertness, temperature control, anxiety, lethargy, or depression [45]. In a high-temperature environment, despite sufficient energy sources, high-intensity or long-duration exercises cause an increase in body temperature, which may lead to changes in the CNS, ultimately resulting in central fatigue [27, 45, 46]. One of these changes involves serotonin, which increases in the body and the brain during exercise, causing muscle fatigue and impairing muscle contraction [47]. The increase in serotonin during sustained exercise occurs as lipids are broken down and converted to free fatty acids for use as an energy source [45]. This increases the concentration of free tryptophan in the blood, which crosses the blood-brain barrier and is used to synthesize serotonin, which contributes to central fatigue during exercise [37]. According to previous studies on the relationship between serotonin and aerobic capacity in a human study, pharmacological treatment with selective serotonin reuptake inhibitors decreased physical performance in a time to reach exhaustion cycling trial, suggesting that activity of the serotonergic system in the brain could modulate fatigue during prolonged exercise [48]. In this study, we found that serotonin levels were reduced by MCE supplementation during exercise. This result suggests that MCE supplementation, which prevents serotonin secretion, may enhance aerobic performance with training.

Serotonin, dopamine, and prolactin have been reported to stimulate and inhibit each other in the brain [49]. In fact, there is a decrease in dopamine when serotonin synthesis and secretion increase [50]. In a fatiguing exercise rat model, decreased serotonin and increased dopamine levels in the brain were observed during prolonged exercise [51]. Dopamine levels decreased in the training phase compared to those in the preparation phase and increased again at rest [52]. The results of this study are consistent with those of previous studies that report an inverse relationship between serotonin and dopamine, which implies that MCE supplementation helps to maintain a balance of vital neurotransmitters for fatigue relief to promote the training effectiveness. In addition to examining the validity of using serotonin and dopamine as indicators of fatigue in high-intensity training, serotonin levels increased more in the training phase than in the preparation phase and remained high during rest [49]. These results demonstrate that serotonin and dopamine are suitable indicators for evaluating the central fatigue that is often associated with mood alterations including depression and anxiety [45, 53]. A systemic in vivo study using specific antagonist compounds tried to elucidate the mechanism of the antidepressant- and anxiolytic-like effects of MCE via interaction with dopaminergic (D_2_ receptor) and serotonergic (5-HT_2_ receptor) mechanisms [54]. Although it remains unclear what physiological mechanisms are responsible for MCE-rescued central fatigue, a plausible candidate may be the MCE-mediated release of neuromodulators including dopamine and serotonin.

Another change seen in central fatigue is in prolactin levels. Prolactin is secreted from the anterior pituitary gland and is increased throughout the body during exercise in a high-temperature environment [55]. Prolactin levels increase in proportion to an increase in body temperature, and prolactin secretion is stimulated by serotonin and inhibited by dopamine [56]. This is also supported by a previous study reporting that prolactin is activated by serotonin secretion [57]. Among the changes in central fatigue symptoms after MCE supplementation, the fact that serotonin levels decreased indicates that MCE may be effective in helping the body sustain exercise. Since increased serotonin levels are the result of an increase in body temperature, it is thought that MCE helped to reduce and maintain body temperature. In addition, dopamine is involved in the inhibition of prolactin [58], and the reduction in prolactin levels following MCE use appears to occur due to increased dopamine levels. It is known that central fatigue during exercise is a complex phenomenon caused by the various neurotransmitters [59], and the interaction between dopamine and serotonin may play an important role in exercise-induced central fatigue [60]. These MCE-induced antifatigue effects may be caused by hormonal balancing among dopamine, serotonin, and prolactin in the central nervous system. Furthermore, our study demonstrates that there is a varying degree of interaction between MCE use and fatigue indicators, suggesting that MCE could inhibit symptoms of central fatigue and thus enable sustained exercise.

There are limitations to this study that should be considered. First, it is difficult to generalize the results because of the small sample size. In fact, the present study is a pilot trial and addresses feasibility issues, such as sample size, resources, and scientific aspects, prior to a future definitive trial to explore the effects of MCE. In addition, in order to confirm the effect of MCE, the subjects were controlled by residence at a camp and nutritional intake analysis to confirm the temporal change of fatigue substances. However, there was a limitation because a control group or placebo group could not be established. Nevertheless, we were able to establish preliminary evidence for the efficacy of MCE as a supplement for high-intensity athletes.

## 5. Conclusions

This pilot study shows that MCE has a positive effect on several peripheral and central fatigue parameters in athletes performing high-intensity, long-duration summer training in a high-temperature environment. In the future, it will be necessary to perform research on the antioxidant activity of MCE, changes in heat and stress-related hormones, and changes in immune function-related hormones, which could explain more clearly the changes observed in fatigue parameters following MCE supplementation. Furthermore, we anticipate research related to MCE's functional role in cognitive abilities that is crucial for high exercise performance.

## Figures and Tables

**Table 1:** Physical characteristics of study participants.

Variable	Content
Age (years)	22.20 ± 0.90
Height (cm)	177.20 ± 6.70
Weight (kg)	70.80 ± 12.30
BMI (kg/m^2^)	22.30 ± 2.90
Careers (years)	5.90 ± 2.70

Values are expressed as mean and standard deviation.

**Table 2:** Changes in nutritional intake according to dietary intake during summer season training before and after MCE supplementation.

Variable	Before experiment	After experiment	*t*-value	*p*-value
Total energy (kcal/day)	1550.91 ± 717.27	1821.95 ± 893.91	-1.468	0.153
Carbohydrate (g/day)	208.22 ± 70.34	240.61 ± 111.43	-1.315	0.199
Lipid (g/day)	50.55 ± 37.26	60.78 ± 42.82	-1.268	0.215
Protein (g/day)	53.10 ± 28.74	59.29 ± 16.05	-1.170	0.251
Fiber (g/day)	16.04 ± 8.34	16.69 ± 8.37	-0.335	0.740
Ash (g/day)	13.17 ± 5.52	15.13 ± 4.32	-1.696	0.101

Values are expressed as mean and standard deviation.

**Table 3:** Ingredients of MCE (g/100 g).

Variable	Content
Natrium	10.40
Vitamin C	6.420
Calorie	0.40
Carbohydrate	0.10

Analyzed by the Korea Food Research Institute and Pukyong National University Food Analysis Center.

**Table 4:** Summer season daily training program.

Weeks	Time	Main exercise	Specific program	Intensity	Frequency
1–4	10 : 00–10 : 10	Warm-up	Dynamic stretching (upper and lower joint body)	40%–60% HRR RPE 12–14	5 times/week
	10 : 10–11 : 00	Aerobic training	Buffy test, arm walking, stairs run, shuttle run (20 m)	70%–90% HRR RPE 15–19 20–30 times/3 set Set rest: 60 s	
	11 : 00–11 : 10	Rest			
	11 : 10–12 : 00	Resistance training	Chest, shoulder, back, arm (biceps, triceps, and wrist), lower body and abdominal	60%–85% 1RM OMNI RES 6–8 15 times/3 set Set rest: 60 s	
	12 : 00–12 : 10	Cool-down	Static stretching (upper and lower joint body)	20%–40% HRR RPE 9–11	
	Free time				
	15 : 00–15 : 10	Warm-up	Tennis court running (3 raps) and dynamic stretching (upper and lower joint body)	40%–60% HRR RPE 12–14	
	15 : 10–16 : 00	Skill-related training	Forehand groundstroke, backhand groundstroke, and running groundstroke	70%–90% HRR RPE 15–19 20 times/3 set Set rest: 60 s	
	16 : 00–16 : 10	Rest			
	16 : 10–17 : 00	Skill-related training	Forehand volley, backhand volley, and jump smashing (connect/rotation)	70%–90% HRR RPE 15–19 30 times/3 set Set rest: 60 s	
	17 : 00–17 : 10	Rest			
	16 : 20–17 : 00	Game	Single game (1 set/6 games)	70%–90% HRR RPE 15–19	
	17 : 00–17 : 10	Cool-down	Tennis court running (2 raps) and static stretching (upper and lower joint body)	20%–60% HRR RPE 10–14	

HRR: heart rate reserve; RPE: rate of perceived exertion; 1RM: one-repetition maximum; RES: resistance scale of perceived exertion.

**Table 5:** Changes in peripheral fatigue parameters during summer season training before and after MCE supplementation.

Variable	Before exercise	During exercise	After exercise	Morning after exercise	*F*	
Ammonia (*μ*g/dL)						
Pre	65.10 ± 15.54	92.80 ± 14.11^##^	103.60 ± 25.37^###^	90.45 ± 19.82^#^	T	6.317^$$^
Post	113.60 ± 36.36	125.70 ± 18.55	135.10 ± 38.61	79.50 ± 27.83^#^	S	24.883^$$^
*t*-value	3.879^∗∗^	4.464^∗∗∗^	2.156^∗^	1.013	T × S	5.250^$$^
Uric acid (mg/dL)						
Pre	6.92 ± 1.71	7.52 ± 1.78^##^	7.85 ± 1.72^##^	7.23 ± 1.52	T	6.003^$^
Post	6.84 ± 1.66	6.95 ± 1.74	6.93 ± 1.65	6.69 ± 1.26	S	3.600
*t*-value	0.106	0.723	1.222	0.464	T × S	4.192

Values are mean and standard deviation. ^∗^*p* < 0.05, ^∗∗^*p* < 0.01, ^∗∗∗^*p* < 0.001 compared to the before-MCE supplementation values, ^#^*p* < 0.05, ^##^*p* < 0.01, ^###^*p* < 0.001 compared to the rest values. T: time effect; S: supplementation effect; T × S: time × supplementation interaction effect, ^$^*p* < 0.05, ^$$^*p* < 0.01.

**Table 6:** Changes in central fatigue parameters during summer season training before and after MCE supplementation.

Variable	Before exercise	During exercise	After exercise	Msorning after exercise	*F*	
Serotonin (ng/mL)						
Pre	122.48 ± 27.54	156.42 ± 38.68^###^	157.79 ± 43.58^##^	137.75 ± 42.19	T	12.791^$$^
Post	114.76 ± 25.30	120.58 ± 24.74^#^	135.59 ± 30.79^###^	118.92 ± 37.85	S	11.117^$$^
*t*-value	0.653	2.468^∗^	1.316	1.051	T × S	3.134^$^
Dopamine (ng/mL)						
Pre	23.54 ± 2.55	21.55 ± 2.99^#^	24.71 ± 4.26	23.03 ± 4.99	T	1.327
Post	24.93 ± 2.93	25.62 ± 3.11	25.76 ± 2.65	23.40 ± 4.54	S	5.186^$^
*t*-value	1.130	2.985^∗^	0.662	-0.174	T × S	0.954
Prolactin (ng/mL)						
Pre	24.53 ± 11.51	29.57 ± 11.81	31.41 ± 20.37	15.73 ± 7.11	T	0.610
Post	18.63 ± 6.58	15.27 ± 5.80^##^	14.21 ± 5.52^#^	19.72 ± 7.19	S	6.830^$^
*t*-value	1.407	3.438^∗∗^	2.577^∗^	1.246	T × S	4.652^$^

Values are mean and standard deviation. ^∗^*p* < 0.05, ^∗∗^*p* < 0.01 compared to the before-MCE supplementation values, ^#^*p* < 0.05, ^##^*p* < 0.01, ^###^*p* < 0.001 compared to the rest values. T: time effect; S: supplementation effect; T × S: time × supplementation interaction effect, ^$^*p* < 0.05, ^$$^*p* < 0.01.

## Data Availability

The data used to support the findings of this study are available from the corresponding author upon request.

## Abbreviation

1RM: one-repetition maximum
ATP: adenosine triphosphate
CNS: central nervous system
HRR: heart rate reserve
MCE: *momordica charantia* extract
RPE: rate of perceived exertion
RES: resistance scale of perceived exertion.

## Additional Points

*Highlights*. Momordica charantia extract (MCE) is an effective supplement in fatigue exposure. MCE increases ammonia levels before, during, and after exercise but speeds recovery. MCE decreases serotonin and prolactin levels during and after exercise. MCE increases dopamine levels during exercise.

## References

[1] Russell M., Benton D., Kingsley M. (2014). Carbohydrate ingestion before and during soccer match play and blood glucose and lactate concentrations. *Journal of Athletic Training*.

[2] Mielgo-Ayuso J., Calleja-Gonzalez J., Del Coso J., Urdampilleta A., León-Guereño P., Fernández-Lázaro D. (2019). Caffeine Supplementation and Physical Performance, Muscle Damage and Perception of Fatigue in Soccer Players: A Systematic Review. *Nutrients*.

[3] Vitale K., Getzin A. (2019). Nutrition and supplement update for the endurance athlete: Review and recommendations. *Nutrients*.

[4] Hsueh C.-F., Wu H.-J., Tsai T.-S., Wu C.-L., Chang C.-K. (2018). The effect of branched-chain amino acids, citrulline, and arginine on high-intensity interval performance in young swimmers. *Nutrients*.

[5] Michalczyk M., Chycki J., Zajac A., Maszczyk A., Zydek G., Langfort J. (2019). Anaerobic performance after a low-carbohydrate diet (LCD) followed by 7 days of carbohydrate loading in male basketball players. *Nutrients*.

[6] Sawka M. N., Burke L. M., Eichner E. R., Maughan R. J., Montain S. J., Stachenfeld N. S. (2007). Exercise and fluid replacement. *Medicine and Science in Sports and Exercise*.

[7] Ha M.-S., Kim J.-H., Ha S.-M., Kim Y.-S., Kim D.-Y. (2019). Positive influence of aqua exercise and burdock extract intake on fitness factors and vascular regulation substances in elderly. *Journal of Clinical Biochemistry and Nutrition*.

[8] Ha M. S., Kim J. H., Kim Y. S., Kim D. Y. (2018). Effects of aquarobic exercise and burdock intake on serum blood lipids and vascular elasticity in Korean elderly women. *Experimental. Gerontology*.

[9] Nyakayiru J., Jonvik K., Trommelen J. (2017). Beetroot juice supplementation improves high-intensity intermittent type exercise performance in trained soccer players. *Nutrients*.

[10] Huang H.-J., Chen S.-L., Chang Y.-T., Chyuan J.-H., Hsieh-Li H. (2018). Administration of Momordica charantia enhances the neuroprotection and reduces the side effects of LiCl in the treatment of Alzheimer’s disease. *Nutrients*.

[11] Jia S., Shen M., Zhang F., Xie J. (2017). Recent Advances in Momordica charantia: Functional Components and Biological Activities. *International Journal of Molecular Sciences*.

[12] Joseph B., Jini D. (2013). Antidiabetic effects of Momordica charantia (bitter melon) and its medicinal potency. *Asian Pacific Journal of Tropical Disease*.

[13] Poovitha S., Siva Sai M., Parani M. (2017). Protein extract from the fruit pulp of Momordica dioica shows anti-diabetic, anti-lipidemic and antioxidant activity in diabetic rats. *Journal of Functional Foods*.

[14] Chen Q., Chan L. L. Y., Li E. T. S. (2003). Bitter Melon (Momordica charantia) reduces adiposity, lowers serum insulin and normalizes glucose tolerance in rats fed a high fat diet. *The Journal of Nutrition*.

[15] Grover J. K., Yadav S. P. (2004). Pharmacological actions and potential uses of Momordica charantia: a review. *Journal of Ethnopharmacology*.

[16] Tan M.-J., Ye J.-M., Turner N. (2008). Antidiabetic Activities of Triterpenoids Isolated from Bitter Melon Associated with Activation of the AMPK Pathway. *Chemistry and Biology*.

[17] Poovitha S., Parani M. (2017). Protein extract from the fruit pulp of Momordica charantia potentiate glucose uptake by up-regulating GLUT4 and AMPK. *Journal of Functional Foods*.

[18] Hsiao C.-Y., Chen Y.-M., Hsu Y.-J., Huang C.-C., Sung H.-C., Chen S.-S. (2017). Supplementation with Hualian No. 4 wild bitter gourd (<i>Momordica charantia</i> Linn. var. <i>abbreviata</i> ser.) extract increases anti-fatigue activities and enhances exercise performance in mice. *The Journal of Veterinary Medical Science*.

[19] Davis J. M., Bailey S. P. (1997). Possible mechanisms of central nervous system fatigue during exercise. *Medicine and Science in Sports and Exercise*.

[20] Finsterer J. (2012). Biomarkers of peripheral muscle fatigue during exercise. *BMC Musculoskeletal Disorders*.

[21] Meeusen R., Roelands B. (2017). Fatigue: is it all neurochemistry?. *European Journal of Sport Science*.

[22] Rudroff T., Kindred J. H., Ketelhut N. B. (2016). Fatigue in multiple sclerosis: misconceptions and future research directions. *Frontiers in Neurology*.

[23] Costanza M., Pedotti R. (2016). Prolactin: friend or foe in central nervous system autoimmune inflammation?. *International Journal of Molecular Sciences*.

[24] Morris G., Berk M., Walder K., Maes M. (2015). Central pathways causing fatigue in neuro-inflammatory and autoimmune illnesses. *BMC Medicine*.

[25] Langeskov-Christensen M., Bisson E. J., Finlayson M. L., Dalgas U. (2017). Potential pathophysiological pathways that can explain the positive effects of exercise on fatigue in multiple sclerosis: a scoping review. *Journal of the Neurological Sciences*.

[26] Quod M. J., Martin D. T., Laursen P. B. (2006). Cooling athletes before competition in the Heat. *Sports Medicine*.

[27] Sawka M. N., Montain S. J., Latzka W. A. (2001). Hydration effects on thermoregulation and performance in the heat. *Comparative Biochemistry and Physiology Part A: Molecular & Integrative Physiology*.

[28] Ely B. R., Cheuvront S. N., Kenefick R. W., Sawka M. N. (2010). Aerobic performance is degraded, despite modest hyperthermia, in hot environments. *Medicine and Science in Sports and Exercise*.

[29] Burnley M., Jones A. M. (2017). Power–duration relationship: physiology, fatigue, and the limits of human performance. *European Journal of Sport Science*.

[30] Ahn M. J., Yuk H. J., Lee H. Y. (2015). Effect of the Enhanced Biological Activities and Reduced Bitter Taste of Bitter Melon(Momordica charantia L.) by Roasting. *Journal of Agriculture & Life Science*.

[31] Kim M.-W. (2013). Effect of bitter melon on plasma blood glucose and cholesterol levels in streptozotocin induced diabetic rats. *Journal of the East Asian Society of Dietary Life*.

[32] Kwak J.-J., Sung G.-D., Baek Y.-H. (2013). Effects of Herbal drinks intake for summer training physical fitness, O2max and E in male college tennis player. *Teacher Education Research*.

[33] Borg G. A. V. (1982). Psychophysical bases of perceived exertion. *Medicine & Science in Sports & Exercise*.

[34] Lagally K. M., Robertson R. J. (2006). Construct validity of the OMNI Resistance Exercise Scale. *Journal of Strength and Conditioning Research*.

[35] Faul F., Erdfelder E., Lang A. G., Buchner A. (2007). G∗Power 3: A flexible statistical power analysis program for the social, behavioral, and biomedical sciences. *Behavior Research Methods*.

[36] Carvalho-Peixoto J., Alves R. C., Cameron L.-C. (2007). Glutamine and carbohydrate supplements reduce ammonemia increase during endurance field exercise. *Applied Physiology, Nutrition, and Metabolism*.

[37] Davis J. M. (2008). Central and peripheral factors in fatigue. *Journal of Sports Sciences*.

[38] Bassini-Cameron A., Monteiro A., Gomes A., Werneck-de-Castro J. P. S., Cameron L. (2008). Glutamine protects against increases in blood ammonia in football players in an exercise intensity-dependent way. *British Journal of Sports Medicine*.

[39] Thenmozhi A. J., Subramanian P. (2011). Antioxidant potential of momordica charantia in ammonium chloride-induced hyperammonemic rats. *Evidence-Based Complementary and Alternative Medicine*.

[40] Chen S., Minegishi Y., Hasumura T., Shimotoyodome A., Ota N. (2020). Involvement of ammonia metabolism in the improvement of endurance performance by tea catechins in mice. *Scientific Reports*.

[41] Lima W. G., Martins-Santos M. E. S., Chaves V. E. (2015). Uric acid as a modulator of glucose and lipid metabolism. *Biochimie*.

[42] Yang Y., Zhou Y., Cheng S., Sun J. L., Yao H., Ma L. (2016). Effect of uric acid on mitochondrial function and oxidative stress in hepatocytes. *Genetics and Molecular Research*.

[43] Green H. J., Fraser I. G. (1988). Differential effects of exercise intensity on serum uric acid concentration. *Medicine and Science in Sports and Exercise*.

[44] Wiecek M., Maciejczyk M., Szymura J., Szygula Z. (2015). Changes in oxidative stress and acid-base balance in men and women following maximal-intensity physical exercise. *Physiological Research*.

[45] Nybo L., Secher N. H. (2004). Cerebral perturbations provoked by prolonged exercise. *Progress in Neurobiology*.

[46] Low D., Cable T., Purvis A. (2007). Exercise thermoregulation and hyperprolactinaemia. *Ergonomics*.

[47] Meeusen R., Watson P., Hasegawa H., Roelands B., Piacentini M. F. (2006). Central Fatigue. *Sports Medicine*.

[48] Teixeira-Coelho F., Uendeles-Pinto J. P., Serafim A. C. A., Wanner S. P. (2014). The paroxetine effect on exercise performance depends on the aerobic capacity of exercising individuals. *Journal of Sports Science and Medicine*.

[49] Van de Kar L. D., Rittenhouse P. A., Li Q., Levy A. D. (1995). Serotonergic regulation of renin and prolactin secretion. *Behavioural Brain Research*.

[50] Cordeiro L. M. S., Rabelo P. C. R., Moraes M. M. (2017). Physical exercise-induced fatigue: the role of serotonergic and dopaminergic systems. *Brazilian Journal of Medical and Biological Research*.

[51] Bailey S. P., Davis J. M., Ahlborn E. N. (1993). Neuroendocrine and substrate responses to altered brain 5-HT activity during prolonged exercise to fatigue. *Journal of Applied Physiology*.

[52] Roelands B., de Koning J., Foster C., Hettinga F., Meeusen R. (2013). Neurophysiological determinants of theoretical concepts and mechanisms Involved in pacing. *Sports Medicine*.

[53] Chaudhuri A., Behan P. O. (2004). Fatigue in neurological disorders. *The Lancet*.

[54] Ishola I., Akinyede A., Sholarin A. (2014). Antidepressant and Anxiolytic Properties of the Methanolic Extract of Momordica charantia Linn (Cucurbitaceae) and its Mechanism of Action. *Drug Research*.

[55] Zhao J., Lai L., Cheung S. S. (2015). Hot environments decrease exercise capacity and elevate multiple neurotransmitters. *Life Sciences*.

[56] Pearce S., Mostyn A., Alves-Guerra M. C. (2003). Prolactin, prolactin receptor and uncoupling proteins during fetal and neonatal development. *Proceedings of the Nutrition Society*.

[57] Fitzgerald P., Dinan T. G. (2008). Prolactin and dopamine: What is the connection? A Review Article. *Journal of Psychopharmacology*.

[58] Bole-Feysot C., Goffin V., Edery M., Binart N., Kelly P. A. (1998). Prolactin (PRL) and its receptor: actions, signal transduction pathways and phenotypes observed in PRL receptor knockout mice. *Endocrine Reviews*.

[59] Meeusen R., Roelands B. (2010). Central fatigue and neurotransmitters, can thermoregulation be manipulated?. *Scandinavian Journal of Medicine & Science in Sports*.

[60] Foley T. E., Fleshner M. (2008). Neuroplasticity of Dopamine Circuits After Exercise: Implications for Central Fatigue. *NeuroMolecular Medicine*.

